# The politics of agricultural policy and nutrition: A case study of Malawi’s Farm Input Subsidy Programme (FISP)

**DOI:** 10.1371/journal.pgph.0002410

**Published:** 2023-10-11

**Authors:** Helen Walls, Deborah Johnston, Mirriam Matita, Tayamika Kamwanja, Richard Smith, Simeon Nanama

**Affiliations:** 1 Department of Global Heath and Development, Faculty of Public Health & Policy, London School of Hygiene & Tropical Medicine, London, United Kingdom; 2 Department of Economics, SOAS University of London, London, United Kingdom; 3 Department of Economics, University of Malawi, Zomba, Malawi; 4 Lilongwe University of Agriculture and Natural Resources, Lilongwe, Malawi; 5 School of Business, University of Leicester, Leicester, United Kingdom; 6 College of Medicine and Health, University of Exeter, Exeter, United Kingdom; 7 United Nations Children’s Fund (Unicef), Abuja, Nigeria; Institute of Public Health Bengaluru, INDIA

## Abstract

The concept of food and nutrition policy has broadened from simply being an aspect of health policy, to policy interventions from across a wide range of sectors, but still with potentially important impact on nutritional outcomes. This wider and more complex conceptualisation involves policy with multiple objectives and stakeholder influences. Thus, it becomes particularly important to understand the dynamics of these policy processes, including policy design and implementation. To add to this literature, we apply the Kaleidoscope Model for understanding policy change in developing country contexts to the case-study of an agricultural input subsidy (AIS) programme in Malawi, the Farm Input Subsidy Programme (FISP), exploring the dynamics of the FISP policy process including nutritional impact. Over a three-month period between 2017 and 2019 we conducted in-depth interviews with key stakeholders at national and district levels, and focus groups with people from rural districts in Malawi. We also undertook a review of literature relating to the political economy of the FISP. We analysed the data thematically, as per the domains of the Kaleidoscope Model. The analysis across the FISP policy process including policy design and implementation highlights how stakeholders’ ideas, interests and influence have shaped the evolution of FISP policy including constraints to policy improvement–and the nutritional impacts of this. This approach extends the literature on the tensions, contradictions and challenges in food and nutrition policy by examining the reasons that these occur in Malawi with the FISP. We also add to the political science and policy analysis literature on policy implementation, extending the concept of veto players to include those targeted by the policy. The findings are important for consideration by policymakers and other stakeholders seeking to address malnutrition in rural, food-insecure populations in Malawi and other low-income settings.

## Background

A considerable body of literature alerts development practitioners to the importance of food security and nutrition for social, health and economic development [1–3]. For example, in low- and middle-income countries, an estimate of between 2% and 11% of gross domestic product (GDP) is lost due to malnutrition, with the cost of iron deficiency at about 4.5% of GDP [4]. In Malawi, the African Union Commission and World Food Programme estimated the cost of malnutrition to be approximately 10% of the country’s annual GDP [5]. Government prioritisation of food and nutrition policy should conceptualise nutrition as beyond just the remit of a health department, given that nutritional outcomes are influenced by the wider social, economic, and political context [6]. As such, improving population nutrition requires engaging with the political economy of food systems [7, 8]. This includes recognition that conceptualisations of (mal)nutrition and its determinants are themselves politicised, with different stakeholders prioritising different factors and aspects of malnutrition for intervention [9–11]. While the concepts of food security, healthy nutrition and food safety are all important to food systems and health outcomes [9], in this study we particularly focus on food security concerned with food availability, access, utilisation and stability as well as aspects of healthy nutrition related to poor dietary diversity and micronutrient deficiency.

Since the early 1990s many African countries have invested in a potentially important upstream policy driver of nutrition, agricultural input subsidy programmes (AISP), to boost agricultural productivity and food security [12, 13]. AISP are grants or loans given to farmers to reduce the cost of acquiring a specific input used in agricultural production (e.g. inorganic fertiliser; hybrid seeds). But there is considerable debate regarding the effectiveness and efficiency of AISP investments, especially in regard to their ability to meet set goals including improving nutrition through diversifying diets [14, 15]. Nutritional status is determined by several factors, including dietary diversity [16, 17], an area potentially affected by AISP. AISP targeting maize, for example, may increase production and consumption of maize and hence reduce intake of nutrient-rich foods or, if maize prices fall, may provide consumers with more real disposable income to spend on other food items [18]. According to Ruel et al. (2010), food price changes in poor countries may prompt people to consume staple foods of different quality or price, alter their overall food intake, switch to nutrient-rich non-staples, and change their consumption of cheaper, high-calorie but low-nutrient foods, all of which contribute to nutritonal outcomes [19]. With AISP in Malawi, national food self-sufficiency in maize has been registered except during periods of weather shocks [20]. Diets, however, have remained undiversified with consumption biased towards maize [21], a crop connected with micronutrient deficiencies [22].

**Fig 1:**
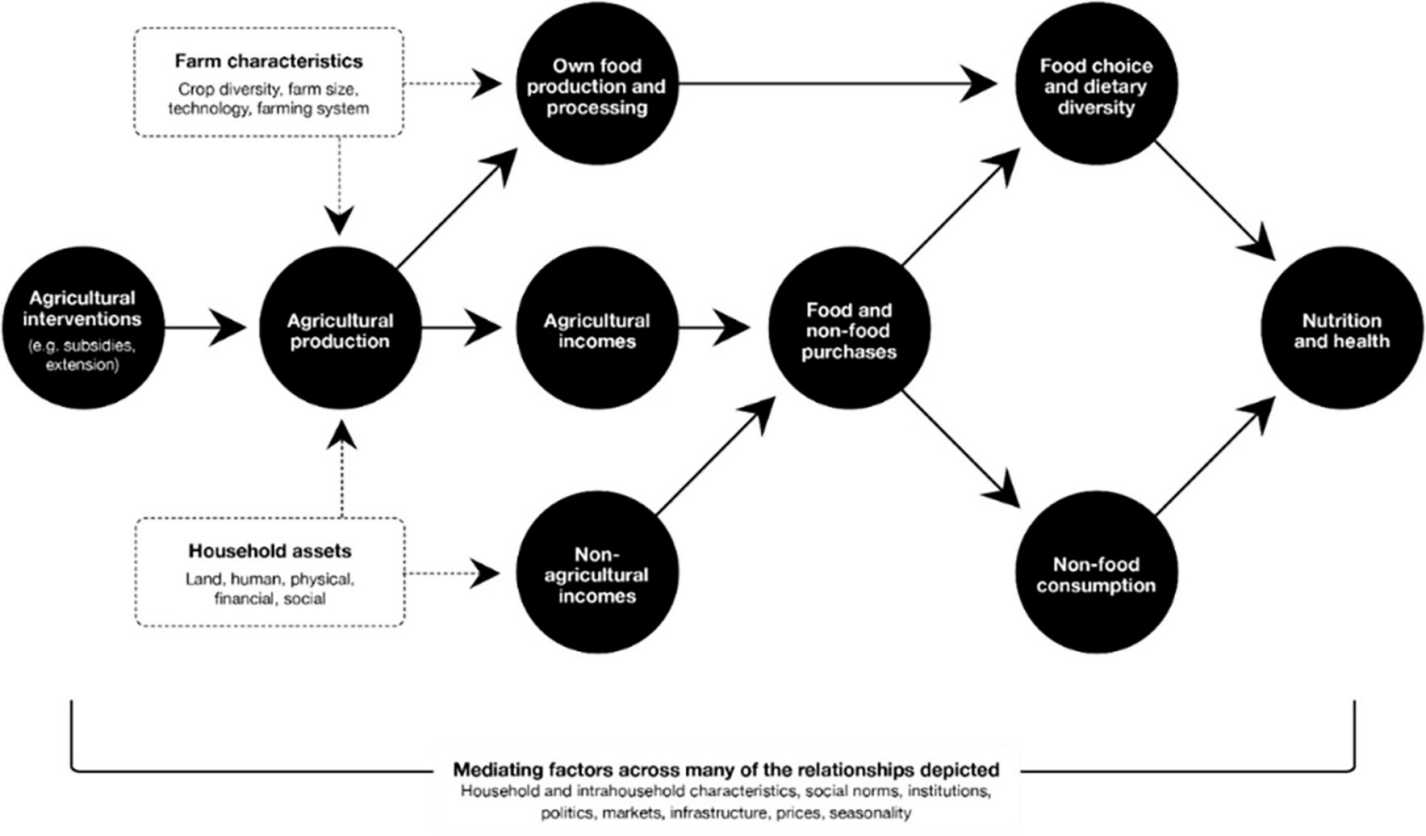
Conceptual framework linking agricultural interventions to nutrition and related health. Source: Matita et al., 2021a.

Fig 1 conceptualizes potential impact pathways from AISP to food choice, dietary diversity, nutrition and related health.

In Malawi, persistent problems with low agricultural productivity, food insecurity, hunger and poor nutritional outcomes have kept the focus of the Malawian government and other stakeholders on the issue of food and nutrition. Alongside various nutritional interventions since the 1970s have been strategies, including AISP, for improving smallholder incomes and achieving household food security led by the agriculture sector [23]. As of 2016/17, almost two thirds of households in Malawi (61%) were considered to have ‘very low food security’, defined as low quality, variety, quantity and frequency of food consumption, and this proportion was even higher (66%) in rural areas [24]. Only one in four children aged 6–23 months met minimum dietary diversity (2015/16 figures) [25]. Although stunting rates have declined (from 47% in 2010 to 37% in 2015/16), they remain high [25].

Malawi’s Farm Input Subsidy Programme (FISP) was one of the most prominent AISP globally [15]. The FISP, implemented 2005–2020, aimed to increase agricultural productivity, household food security, and the incomes of resource-constrained households [26]. While its size and reach varied over time, it made up a large proportion–up to 75%–of the government’s agricultural budget [13, 27]. In 2008/09, the fertiliser and seed coupons issued were together worth around US$275 million, with each fertiliser coupon’s value greater than 10% of annual household income for more than 40% of the population [26]. In 2010/11 the programme directly benefitted 79% of farming households [26]. The FISP initially provided subsidies on improved maize seeds and fertiliser only, but from 2008 also included legume seeds [26]. In 2020, the new Malawi Government replaced the FISP with the Affordable Inputs Program (AIP), which extends the subsidy on maize seed and fertiliser to sorghum and rice seed, and is more universal, with all smallholder farmers eligible, rather than being targeted [28].

Of the few studies undertaken of FISP impact on dietary outcomes, some show little impact while others have mixed results [15, 29–32]–similar to studies of large-scale AISP impact on dietary outcomes from countries other than Malawi [33–35]. Previous studies of the FISP have identified a range of policy implementation challenges related to programme objectives and targeting, capture of benefits by elites and well-connected persons, sale of coupons or gifting of coupons, and late delivery of inputs, that would likely dilute any possible programme effect on diet and nutrition [31]. However, there has been little research of the impact of the FISP and its political economy on population nutrition [15, 20, 36, 37]. A review of Walls et al. (2018) also found a lack of focus on the impact of AISP on diets more broadly in existing literature. Chinsinga (2012), Mason et al. (2013) and Mdee et al. (2020) discuss how AISP are at the core of political sentiment in Malawi as well as neighbouring Zambia, relating to food security and legitimacy for voters and political office holders, respectively [20, 36–38]. In turn, agricultural policy processes are conflated with political objectives in Malawi. Whilst existing literature discusses the policy process including issues with policy implementation and other political economy factors that determine the success of AISP there is limited emphasis on nutritional outcomes including how the FISP could improve dietary diversity.

Thus this study builds upon existing work on the political economy of Malawi’s FISP to understand its potential contribution to address malnutrition and reflect on its recent replacement by the AIP. It utilises the Kaleidoscope Model for understanding policy processes and the political economy factors shaping them [39], in particular for understanding the ‘gap’ between expectations on the part of policy makers and the on-the-ground implementation.

### Conceptual approach taken in this study

Recognition of a ‘gap’ between policy expectations and implementation dates back to the 1970s [40], with some scholars focusing on a ‘top-down’ approach to policy implementation, and others on a ‘bottom-up’ approach. The top-down approach is concerned with the mechanisms by which the intentions of policymakers can be most effectively translated into action [41–43]. The bottom-up approach considers the influence of street-level bureaucrats (front-line staff administering benefits, social workers, teachers, local government officials, doctors and nurses) on the implementation of the policy [44]. Front-line policy implementers are seen as having considerable autonomy from this bottom-up perspective, as opposed to passively actioning decisions from above [45]. Numerous syntheses of the two approaches have emerged that recognise the importance of both elite-level policymakers and front-line implementers [46–48].

**Fig 2:**
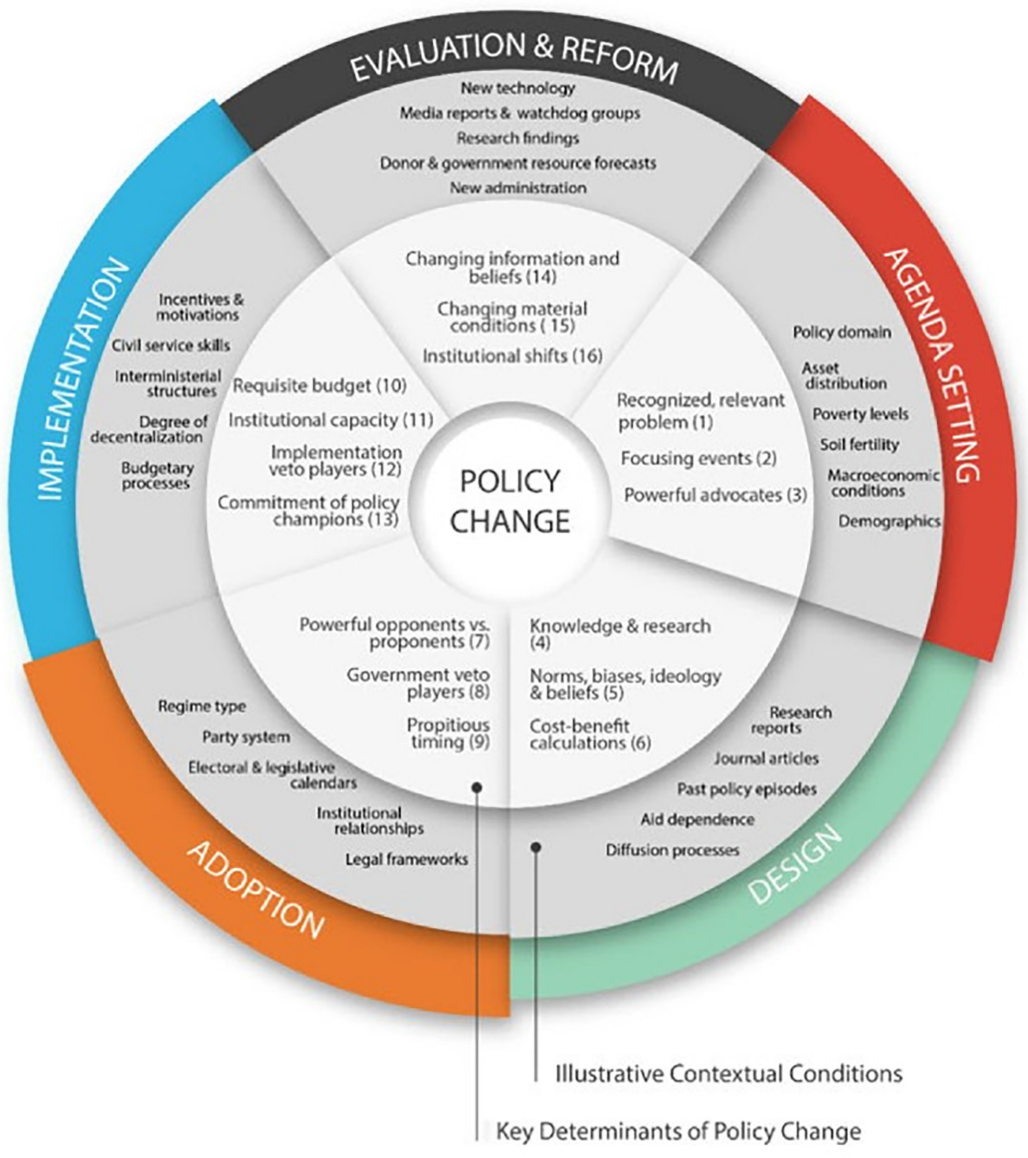
The Kaleidoscope Model of policy change. Source: Resnick et al., 2018.

The ‘Kaleidoscope Model’ (KM) (Fig 2) sets out policy change more holistically, relating to the full policy process (not just implementation) and its underlying factors in a developing country context [39]. Although the authors acknowledge that policy processes are in reality iterative and non-linear, the KM is organised by the five stages of the policy cycle (agenda setting, design, adoption, implementation, and evaluation and reform) which are often considered a useful heuristic for understanding the policy process.

In this study, we draw on the KM to examine the gap between FISP policy expectations and on-the-ground implementation. We do so by examining all categories (domains and sub-domains) of the KM, not simply those in regard to policy implementation, or policy design and implementation. Although useful conceptually to understand the policy process in separate stages, with policy implementation being one of these stages, in practise the policy process is far more complex. Drawing on recent literature critiquing policy analysis methods for often being too reductionist [49–51], in this study we chose to take a broader more contextual analysis of the policy process, with the KM allowing us to capture this complexity while still allowing for research tractibility. This means considering all the policy process stages rather than just one stage, whilst being mindful that the categorisations are somewhat arbitrary, with overlap between the different categories. Taking this broader perspective achieves a more contextual analysis of the FISP, in terms of its expectations and on-the-ground implementation particularly in regard to addressing malnutrition.

## Methods

This study was nested in a larger work programme examining FISP impact on dietary diversity and the context for this impact [citations redacted]. The study’s regional focus was Malawi’s Lilongwe and Phalombe Districts, chosen for their different contexts. Lilongwe District, home to the capital city of Malawi, has a farming system dominated by maize cultivation. Phalombe District is situated in a remote region in the southeast of the country and has a more mixed farming system [28, 52, 53]. In each of the two districts, one Traditional Authority (TA) was randomly sampled from a list of areas included in the FISP evaluation studies, and four enumeration areas in each TA were selected for study.

To examine the perspectives of stakeholders, we undertook focus group discussions (FGDs) and key informant interviews over a three-month period during 2017–19. We conducted 24 in-depth semi-structured interviews with 25 key informants at national and district levels, and 16 FGDs in two enumeration areas of each TA, both in the post-harvest (May 2017) and lean season (February/March 2018)–given the impact of seasonal variation on food prices and diets. Stakeholder groups were purposively sampled using an initial stakeholder analysis, based on their interest in promoting agriculture and/or nutrition, their district responsibilities, or being part of the rural communities. Additionally, respondent suggestions from earlier interviews were followed up (snowball sampling). We attempted to recruit government stakeholders in senior roles, but this was challenging, and our interviewees are from a range of levels of seniority. Table 1 presents information regarding the interviews and FGDs conducted.

**Table 1:** Interviews conducted for each stakeholder group.

Stakeholder group		Number
Central government	Ministry of Health; Ministry of Agriculture, Irrigation and Water Development	6 interviews, with 7 individuals
Regional government	Lilongwe and Phalombe District Councils	7 interviews, with 7 individuals
Non-governmental	One Fund Acre, National Smallholder Farmers Association of Malawi (NASFAM), Civil Society Agriculture Network (CISANET), German development agency Deutsche Gesellschaft für Internationale Zusammenarbeit (GIZ), The Hunger Project	5 interviews, with 5 individuals
Community	Village chiefs and village residents of rural areas of Lilongwe and Phalombe Districts	6 interviews with 6 chiefs, 16 focus groups with other village residents*

* FGDs were conducted in May 2017 and February/March 2018 (two with men, two with women, in the two districts, at each time point).

Interviews lasted an average of 60 minutes, with most around 45 minutes. FGDs lasted around one hour. Interviews were generally conducted in English, except those in the villages, which were undertaken in the local language, Chichewa. Where possible, we audio-recorded interviews otherwise, detailed notes were taken in English by the interviewer. With FGDs, one of two data collectors took notes in English. We conducted interviews until saturation had been reached. Interview guides for in-depth interviews and FGDs covered questions relating to participants’ view of the FISP and its aims, how FISP policy is made, key actors and stakeholders involved with the FISP and their role/influence, the impact of the FISP on nutrition, and the wider context (political, economic, and social) for the FISP. Interview guides were developed, translated, and amended with the support of data collectors, and piloted prior to use. Respondents provided informed consent, mostly in written form and for some FGD participants provided as an ink thumb print. The study investigators had access to information that could identify individual participants during the analysis stage following data collection, but all analysis is reported in a way that individual participants cannot be identified. The research was conducted with ethical approval from [redacted].

We transcribed and analysed the recorded interviews using NVivo10 data analysis software. Interview transcripts were read independently by two investigators (HW, DJ), and potential codes for analysis identified. Following this, codes were agreed between the two investigators before formal coding was undertaken in NVivo. Then, we analysed the data thematically, identifying themes relating specifically to the KM of policy change. We summarise in Table 2 the key KM components, which provide the structure for our presentation of results.

**Table 2:** Key categories of analysis drawn from the Kaleidoscope Model of policy change.

Agenda setting	Recognised, relevant problem
	Focusing events
	Powerful advocates
Policy design	Knowledge and research
	Norms, biases, ideologies and beliefs
	Cost-benefit calculations
Adoption	Powerful opponents versus proponents
	Government veto players
	Propitious timing
Policy implementation	Requisite budget
	Institutional capacity
	Implementation veto players
	Commitment of policy champions
Evaluation and reform	Changing information and beliefs
	Changing material circumstances
	Institutional shifts

In addition to interviews and FGDs, we also undertook a review of literature relating to FISP political economy, and synthesised information from different data sources, following the KM domains.

## Results

### Agenda setting

The severe nature of food insecurity and malnutrition is well recognised by stakeholders in Malawi, including from the highest levels of government. This reflects not only the country’s food crises of the 2001/02 and 2004/05 agricultural seasons which affected 3.2 and 4 million people respectively, but also a long history of food shortages and pervasive food insecurity [36, 54, 55].

Governmental respondents acknowledged significant problems with food and nutrition security in Malawi, sometimes citing challenging statistics, disparities within the country, and described how nutrition was high on the political agenda. A respondent from a donor agency spoke about how national stunting statistics conceal large regional inequities.

“*There has been reduction [in stunting levels in Malawi] but we have not reached the minimum standards*. *If you look at 37% it is still high and 37% you are looking at it to be national but if you go dig into districts you find that levels are still high in other districts like Mangochi*. *If you start isolating*, *you find that malnutrition levels are still high though strides are being made*.*” (KII 22*, *non-governmental organisation)*

The issues of food insecurity and malnutrition appear to be a high priority for the Malawian government. Malawi is considered to have a proactive National Nutrition Policy and underlying cross-sector institutional framework [23]. In 2006 the Department of Nutrition HIV and AIDS (DNH) was moved from the Ministry of Health (MoH) and placed under the Office of the President and Cabinet (OPC), but later returned to the MoH. Respondents had different views on the institutional set up for addressing nutrition and what it means in terms of government commitment to the issue. A MoH respondent described how this high political prioritisation of nutrition remains, despite the institutional restructuring. In contrast, a respondent from a donor agency suggested that the restructuring was accompanied by some deprioritisation of the issues on the political agenda. Indeed, in Malawi 2063, a long-term national multisectoral vision of Malawi for the period 2020–2063, health and nutrition are only acknowledged as an enabler to enhancing human capacity for achieving the three pillars of agricultural productivity and commercialisation, industrialisation, and urbanisation for creating an inclusively wealthy and self-reliant nation [21]. The National Multi-Sector Nutrition Policy (2017–2021) however, emphasizes the importance of adequate nourishment of the population for the achievement of development goals [56].

“*In Malawi*, *nutrition is one of the highest on the political agenda and [several years ago]*, *the minister responsible for nutrition was His Excellency*, *the President… and then it was under the OPC*.*” (KII 18*, *Ministry of Health)*

“*When the DNH was within the OPC*, *they had a higher-level mandate*. *They were placed at a higher office to coordinate the efforts*. *But now they have gone back to MoH*. *So*, *with the coordinating department being within a line Ministry*, *it makes it difficult for them… to coordinate the other ministries; because it is like they are at the same level*. *But when they were at OPC it was easier for them*. *That is one of the major drawbacks*. *Because you need a higher office to bring together the different ministries*, *but discussions are under way on how best to ensure that DNH does not lose its coordinating power*. *Actually*, *we need a Coordinating Office*, *we cannot be talking about multi-sectoral collaboration without a coordinating office*. *So DNH is there but its power was reduced*.*” (KII 22*, *non-governmental organisation)*

Community participants also recognised the intersection of seasonality in agricultural production and food and nutrition security [57, 58]. In our FGDs village women described how food insecurity drives their seasonal fluctuations in body weight.

“*Like this time*, *we don’t have enough food we can eat once a day*, *so our bodies tend to get smaller and with farming it just gets worse*.*” (FGD 10*, *women*, *Lilongwe District*, *lean season)*

Village respondents also described diets in rural Malawi as limited in range, with people also not having enough to eat. Most commonly eaten was *nsima* (a maize flour porridge, and the staple food in Malawi), sometimes with a leafy relish made from Indian mustard or pumpkin leaves. If breakfast was eaten, this might be a porridge or *matsukwa* (leftover water after soaked/pounded maize removed). Lunch was often delayed until mid-afternoon so people didn’t feel too hungry before evening. Respondents reported seldom eating meat due to its unaffordability. Chicken was only eaten on celebrations like Independence Day, and egg consumption only in October when it is too hot for chicks to survive. Respondents described how when particularly low on food and having to further ration intake, they choose to eat in the evening over the morning. This way, they could wake up with the strength to go back into the field.

“*To be honest in this area if we have eaten goat meat it means the goat has been killed because it was sick otherwise there is no way one would just kill goat for the sake of eating*. *Not possible*.*” (FGD 13*, *men*, *Phalombe District*, *lean season)*

Thus, the severe nature of these issues in Malawi is widely recognised. There is considerable information elsewhere on how poor people of low-income countries facing considerable seasonality in food prices cope with food insecurity, including in Malawi [59–62].

We identified different stakeholder views on the causes of food and nutrition insecurity. While district and national level stakeholders described poor education and insufficient dietary knowledge or narrow food preferences as the cause of limited diets, village respondents in contrast described the cause as limited resources and food unaffordability. They often linked their inability to afford a diverse diet to the low prices they received for the crops they grew, and sometimes also due to limited availability.

“*[A diverse diet means] vegetables*, *meat*, *having tea in the morning and afternoon as well as eating fruits*. *[Everyone laughs]*. *For us we don’t because we cannot manage*. *It is because some of the foods are expensive*.*” (FGD 10*, *women*, *Lilongwe District*, *lean season)*

“*We eat just to ease hunger*. *We do not have choices of food for there are limited foods available here*. *We only have nsima available here*. *It’s hard to sell even one bag of maize to buy other foods like chips or meat*.*” (FGD 1*, *men*, *Lilongwe District*, *post-harvest season)*

### Policy design

The problematic nature of malnutrition in Malawi was generally well recognised amongst stakeholder groups. However, whilst government stakeholders, aware of Malawi’s poor food and nutritional statistics, sometimes alluded to its consequences for development, other respondents described a lack of understanding (amongst other groups, such as ‘people’ in a general sense) of the consequences of malnutrition.

“*…if one is malnourished it takes time to see the effects*. *People do not understand why people are mentally retarded*, *the cognitive development*, *and you look at our heights*, *we don’t relate it to nutrition we just say*, *‘ok this is normal’ … in the long run the effects of not having a diversified diet*, *the quality of your diet*, *are not shown right there*, *but with the passage of time*.*” (KII 16*, *NGO respondent)*

Reflecting the wider academic and policy debates on the issue, there was little agreement amongst stakeholder groups about how to go about addressing the problem. Policy scholars have recognised that policy choices and decisions are rarely shaped solely by empirical research and evidence. Rather, policy decisions are to a significant extent shaped by the structure and interests of stakeholder institutions including political institutions, and how issues are framed and understood by them [63–65]. For example, divergent views on what causes food and nutrition insecurity and different stakeholder interests in the issue can complicate the development of effective solutions. When questioned as to why FISP had not made a larger impact on food security and diets, the civil servants in our study usually attributed this to individual factors–people’s inflexible food preferences or lack of understanding of how to cook nutritious foods. The civil servants’ proposed solutions were usually about supporting better food choices and dietary practises through nutrition extension activities including education on food preparation.

“*Most of [the reason for malnutrition] is lack of knowledge and skills*… *In some communities when you go there*, *in my experience*, *they may have the things*, *but they don’t know how to combine the foods so that they do not get malnourished*.*” (KII 20*, *Ministry of Health)*

“*We have not had enough education provided to the people in the villages how to prepare meals for their people which are nutritious food*.*” (KII 09*, *District council)*.

However, the village respondents spoke about their diets being shaped by the unaffordability of diverse, healthy foods rather than being due to their choice. The ‘inflexibility’ in Malawian’s food preferences (away from maize) proposed above by civil servants has been described as a familiar trope amongst a variety of actors [66, 67]. Our findings here reflect the alternate view that the dominance of maize in Malawian diets, as also described in other studies [18, 68, 69], is reflective of the wider context of poverty and food insecurity experienced by this population. The differences in stakeholder perspectives on the cause of lack of impact of the FISP on food and nutrition security displays a disconnect on the part of policy makers from the lives of ordinary people, a finding hardly limited to policymakers in Malawi [70, 71], and is a possible reason for policy inadequacy.

“*[A man from the government who visits the village to give health advice] tells us the foods that we need to eat but due to poverty we are unable to get such foods*. *So*, *we just listen to him*… *but we know we cannot afford to eat such foods*. *For example*, *to balance our diets [we] need to eat food from the 6 groups The government of Malawi nutrition education largely promotes six food groups namely*, *staples*, *vegetables*, *legumes & nuts*, *fats & oils*, *animal & animal products*, *myself I cannot afford now I am just waiting to eat nsima after that then that is it*.*” (FGD 11*, *men*, *Lilongwe District*, *lean season)*

“*We hear [that a healthy diet includes] beans*, *meat*, *fish*, *pigeon peas but we don’t eat that people just eat nsima and relish that is all*… *Some of us we don’t even know what cabbage is*. *We also don’t know what carrot is*. *The problem is we don’t have money so we can’t buy*.*” (FGD 13*, *men*, *Phalombe District*, *lean season)*

The FISP’s introduction was clearly not the result of donor influence but rather a response to the 2005 food crisis [72]. Donor organisations had been opponents of subsidy introduction citing distortions to the commercial fertiliser market and limited fiscal space among other reasons [72, 73]. However, such concerns about the lack of sustainability of subsidies provided by governments, underpinned by the neoliberal paradigm shaping the economic liberalisation of the 1980/90s supported by World Bank and International Monetary Fund [9, 74], were later alleviated by the programme’s apparent success [36]. Thus, whilst the donor community eventually supported the programme and influenced reforms, the reasons for the FISP as the chosen policy instrument for addressing dietary-related concerns lie elsewhere.

The FISP’s primary aims were to address national food security and smallholder farmer incomes through improving agricultural productivity [13, 75]–not nutrition or dietary diversity. This is exemplified by the FISP having a focus on subsidies for maize production, with legume seeds (groundnuts, beans, soy beans, pigeon peas) only being added at a later stage (2008), to address soil health through nitrogen fixing and improve nutrition through dietary diversification based on own-farm production [26, 28]. The inclusion of legumes has been to be associated with increases in crop diversification among FISP beneficiaries [76] and also dietary diversity [33, 77, 78], with important caveats as discussed elsewhere [32]. However, the provision of legume seeds has not been consistent, for instance in 2013 due to fiscal distress legume seeds were not made available to farmers. Personal communication with a government official in the Ministry of Agriculture. Furthermore, the successor programme to FISP, the AIP, completely removed subsidies for legume seeds. This development may reinforce the dominant view that maize equals food security, but it could also maintain active stakeholder investments in maize seed as opposed to other cereals and legumes [77]. Whilst Nkhoma (2019) noted that the existing political environment may be the cause of policy oscillations [78], it could as well be the case that any nutritional impacts and improvements to dietary diversity were secondary to the economic and food-security objectives. There are three reasons for this.

First, there is a possible tension between a nutrition policy narrative from the international community and the domestic food security narrative. As Harris (2019) described, nutrition policy in Zambia is split between an international coalition promoting action on child stunting, and a national coalition focused on food security and hunger. Likewise, in Malawi food security is a top priority of government action whilst nutrition is largely considered a health issue [79, 80]. These views are reinforced at grassroots level with a narrative that considers maize security as synonymous with food security [81]. As is well known, addressing hunger and energy requirements is important but insufficient for improving nutritional outcomes [82]. Thus, addressing staple-crop production, potentially through a programme such as the FISP, will not necessarily result in improved nutritional outcomes–but policy-makers are often unaware of the differences/tensions/synergies between these different aspects of malnutrition [9].

Second, the prioritisation of non-nutrition aspects of food security may reflect food security’s perceived strategic importance to national security [9, 32].

Third, and relatedly, there appears to be a prioritisation of addressing food insecurity over nutritional concerns in the context of ongoing food shortages [9, 32] including among the populous in terms of food preferences and cropping pattern choices [83–85], as has also been described in Zambia(10). These tensions are exemplified in the next quote.

“*Agriculture is a priority for the government*, *so resources are pumped into agriculture it is also kind of political*. *If you want more votes then*, *feed the people*, *give them food*. *Whatever kind of food you are giving them [it doesn’t matter] but so long as their tummies are filled*, *nobody is worried*. *But if you look at domestic financing when it comes to nutrition*, *it is quite a challenge*.*” (KII 16*, *NGO respondent)*.

Food shortages and hunger have long been sensitive political issues in Malawi. Malawi’s history of food shortages, in particular the 2001/02 food crisis, set the stage for the country’s May 2004 election–and the subsequent 2004/05 introduction of the FISP–with food security high on the agenda of politicians, policymakers, village chiefs and in media reporting. All major political parties supported fertiliser subsidies as the most feasible approach to addressing food shortages and hunger [27]. The dominant narrative was that hunger and food crises are best addressed by agricultural subsidies to support farmers, with a focus on maize and tobacco–and that through this, Malawi would become food secure rather than being reliant on imports [27].

In fact, Malawi’s agricultural sector has not been without some form of input support since the mid-1970s [86, 87], and AISPs have major political and economic significance in Malawi [36]. This prompted Smale (1995) and others to describe how maize is central to life and politics in Malawi [20, 88]. According to Chinsinga (2012) and Nkhoma (2018), as a result, it is impossible for any Malawian government to ignore agricultural subsidies [20, 72]. The FISP was a large subsidy programme benefitting a larger proportion of the voting population than previous subsidy programmes in Malawi [20]. Chinsinga and Poulton (2014) argue that this broad-based nature can be explained by the concurrent political insecurity faced by the late President Mutharika from 2005–09 [36].

Dorward et al. (2008) and Chirwa et al. (2011) considered the FISP as having the potential to alleviate the constraints that have locked Malawi in this low-maize-productivity trap. Chinsinga (2002) describes the role of unstable maize prices in this productivity trap. First, the fear of low maize prices makes it less attractive for potential maize surplus producers to invest in maize production. At the same time, the fear of high maize prices forces maize-deficit farmers to continue to grow maize even though they cannot afford improved seed and fertilizer. With 60% of Malawi’s smallholder farmers as net buyers of maize and only 10% as net sellers, high maize prices are described as a double-edged sword. Consequently, Dorward and Chirwa have described the role of FISP in reducing agricultural input prices can improve both the affordability and profitability of maize, thus benefiting both net producers and net buyers [89]. With sustained investment, in theory the FISP allows the poorest farmers to eventually diversify their livelihood portfolios beyond growing predominantly maize. In practice, however, the FISP coupled with other policies for example addressing maize prices has failed to support clear livelihood changes and movement out of poverty [90, 91].

Respondents gave similar responses about the aims of the FISP to increase agricultural productivity, although government and NGO stakeholders also tended to emphasis improvements in household food security. However differences widened when we asked what the aims of the FISP *should* be. Respondents from district councils, government ministries and from non-governmental organisations often held sharply different views about FISP objectives particualr as they relate to the issue of targeting and distribution of benefits. To provide context, the literature on subsidies suggests that in principle subsidies should target farmers not already purchasing inputs commercially so as to not crowd-out commercial supply [92, 93].

Regarding the comments about government support for a universal programme as reported by Chinsinga (2012), we identified a tension amongst the views of government stakeholders. In keeping with prior literature documenting an interest among some stakeholders to target more ‘productive’ farmers [72, 78], our government stakeholders frequently expressed a desire to target ‘productive’ farmers rather than something more universal. They suggested that issues of poverty and food insecurity could be addressed directly through other types of social support consistent with prior studies [72, 94–98].

*“I would rather target people who seem to be productive… you are much better off if you gave this to productive people and you leave the rest into safety nets… This programme [the FISP] should be a production investment programme.” (KII 14, Ministry of Agriculture)*

When the FISP was introduced in 2005, guidelines for identifying beneficiaries were unclear. Targeting was decentralised, with village leaders allocating FISP coupons within their communities, and often favouring kin[99, 100]. Over time, targeting criteria were developed, but in practice, often not implemented and frequently challenged in communities. Later, beneficiary selection was undertaken centrally [72, 101]. However, the politics of targeting in terms of criteria and patronage remained [13, 94, 100, 102]. Several studies have described how the FISP’s universal approach to targeting resulted in crowd-out of the private sector [38, 92, 103–105]–with evidence suggesting it considerably reduced farmers’ commercial fertiliser purchases, particularly amongst the wealthiest farmers [92]. Furthermore, wealthier households, and male-headed households, have been more likely to receive coupons [103, 106–108].

From speaking to national-level respondents, we identified a tension between a targeted subsidy programme for productive farmers, which would favour elites, and keeping the majority of the population appeased through a universal programme–a tension that others have also reported [13, 27, 109]. For some respondents, the FISP is rightly a social assistance programme with the aim of targeting all smallholder farmers–or, the poorest farmers. For others, whilst they said that the programme is not designed to target only productive farmers, they believe that such an approach would improve the programme’s efficiency.

### Policy adoption

Several events, and a history of food shortages, had come together to galvanise political support for FISP implementation. Chinsinga (2012) has described how the historical crises in agricultural productivity–largely for maize–are attributed to a combination of: collapse of smallholder farmer credit clubs, liberalisation of agricultural markets in 1980s/90s, which resulted in removal of subsidies on fertiliser, seeds and credit and the hardship caused by the civil war in Mozambique of the 1990s which led to an influx of Mozambiquan refugees; and adverse weather events [36, 74, 110]. Over this time, Malawi shifted from being nationally self-sufficient in maize in non-drought years to being in food deficit and dependent on food imports [66].

Despite these catalysing events, following the 2004 election the government hesitated to implement the FISP [27]. This may have been due to donor warnings that implementation of a subsidy programme would jeopardize prospects of qualifying for debt relief through the implementation of the Malawi Poverty Reduction Strategy (MPRS). As Chinsinga (2007) describes, the FISP’s introduction as a potential solution to Malawi’s crisis in maize productivity happened precisely when the first-generation poverty reductions strategy papers were being reviewed. These documents emphasise domestic ownership as important for sustainable development.

However, with a new food crisis in the 2004/05 agricultural season, affecting 4 million people, and further changes to the political landscape, it soon became difficult for the government to resist implementing the fertiliser programme. The opposition became dominant in Parliament following the decision of the President at the time to form his own political party, after falling out with a former President [111]. The intention of government had been to introduce a AISP targeting only maize [27], however as a minority-led government, it succumbed to sustained opposition pressure to introduce FISP covering both maize and tobacco.

The FISP was therefore implemented in the 2005/6 agricultural season, targeting maize and tobacco [112]. Donors and some local fiscal conservatives strongly opposed its introduction on the basis it ran counter to economic liberalisation efforts, and placed an unsustainable fiscal burden on the state [27, 111]. But the critics largely changed their views following the FISP’s success in the 2005/06 agricultural season [27], when Malawi achieved its biggest-ever maize harvest of 2.6 million metric tonnes, at least 0.5 million tonnes more than the country’s annual requirements, and became an exporter of its maize surplus [36, 89, 111, 113].

### Policy implementation

Following implementation in the face of strong opposition, the FISP largely held the commitment of policy champions notwithstanding being central to some important policy debates both locally and internationally [78]. These debates include in regard to design and implementation efficiencies, fiscal unsustainability as well as allocative inefficiencies within the Ministry of Agriculture, Irrigation and Water Development (MoAIWD) as well as more broadly in the context of limited government financial resources [78]. The FISP accounted for over two-thirds of the financial resources of the MoAIWD and over 50% of total (public) agriculture sector spending between 2007/08 and 2011/12 [78]. Its replacement from 2020, the AIP, takes a similar form. Given the importance to the government of agricultural subsidies for garnering rural support, the government has been described as willing to cling to such a programme, regardless of its merits [20].

Input distribution was undertaken by two government agencies, the Agricultural Development and Marketing Corporation (ADMARC) and the Smallholder Farmer Revolving Fund of Malawi (SFRFM). Private-sector companies were involved in the procurement of fertilizer for the programme alongside SFFRFM through a competitive tendering process. However, tensions arose between the government and donors, with donors arguing for increased involvement of the private sector to facilitate diversification of the programme beyond just maize and tobacco, something considered likely to stimulate sustainable private-sector growth [20, 114]. The government initially wanted to take responsibility for the programme in the aftermath of the 2004/05 food crisis, and to shore up support for the minority ruling Democratic Progressive Party (DPP). The government also recognised the need to partner with the private sector, but had concerns relating to seed quality, the inability to reach remote rural areas not served by the government, and the possibility of unsold government stocks [114]. As a result of this pressure and offers of support to mitigate concerns from the UK Department of International Development, a compromise was reached. The government initially agreed to private-sector involvement in the procurement and distribution of fertilizer and seed, although private-sector involvement in the distribution was subsequently discontinued. Chinsinga (2012b) describes how the government holding onto fertiliser distribution, with the private sector involved in procurement, reflects that fertiliser distribution provides politicians with significant opportunities for rent seeking [20]. The private sector was later involved in direct retailing of fertiliser in selected districts in 2015/16 season as part of the FISP reforms [115]. In 2008, the government also included legumes in the FISP, in an effort to diversify away from maize, improving soil fertility, and boosting farmer income and nutrition [20].

Whilst focused on programme delivery, the government was simultaneously courting chiefs, important players in Malawi’s politics [116], as a strategy for improving government support [117]. Several significant inducements were targeted at the chiefs [20]. Studies analysing the FISP in terms of the theory of rents have shown that districts where a the political party in power received majority of the votes had higher fertiliser voucher distribution [13, 36]; also evident in Zambia [118].

From an international perspective, Malawi’s apparent success with the FISP was due to the country having successfully pioneered the implementation of a ‘smart’ subsidy [26]. Subsidies are considered ‘smart’ if they are part of a broader productivity-enhancement programme, have a clear exit strategy, and are targeted at helping agents overcome market failure [119]. Thus, smart subsidies provide subsidised goods/services designed both to promote private sector input distribution and market development and to address poverty [120]. The extent to which the FISP achieved the precincts of a smart subsidy have however, been challenged in scholarly work [121–126].

Our survey respondents also identified several policy implementation issues and expressed mixed views on the impact of FISP on beneficiaries. Respondents’ frustrations/reservations about the FISP–and reasons provided for why it does not result in improvements to agricultural productivity and nutrition–were particularly about poor targeting of beneficiaries, perceived lack of coupons, delayed coupon distribution, policy coordination problems between stakeholder groups, and coupon sharing and selling-on amongst those in targeted communities.

“*Sometimes beneficiaries may not have bumper yields as the inputs came late*.*” (FGD 2 of men*, *Lilongwe District*, *post-harvest season)*

“*The final register for the beneficiaries is now produced by the agriculture [sector] rather than the village*. *The number of beneficiaries has been reduced by almost 40% in my village*.*” (KII 02*, *village head*, *Phalombe District)*

Despite changes to FISP targeting, leading to beneficiaries being chosen at national level rather than by village chiefs, respondents described how the system still creates problems for the chiefs and their communities.***“****What we have is those that are close to the chiefs are the ones benefitting*.*” (KII 23*, *non-governmental organisation)*

Village respondents described the jealousy felt if they themselves were not recipients. They also explained how the FISP brings conflict and even hatred amongst them, especially to the village chief. Village chiefs, often supportive of the programme, also described the problems it brings to them and their communities.

“*A lot of chiefs have died because of conflicts emanating from coupons*. *There are always conflicts due to shortage of coupons*. *Even the relatives of chiefs have been hurt because people think that they are hiding coupons*, *however the problem is with the government*.*” (FGD 11 of men*, *Lilongwe District*, *post-harvest season)*

**“***Ourselves as Chiefs we have problems with this program… When these coupons are not enough*, *people turn against us and the questions we normally get are ‘do you think I am rich*?*’ So normally the Chiefs are in trouble… There are always conflicts*.*” (KII 5*, *village head*, *Lilongwe)*

Respondents also often spoke about the selling-on of coupons to wealthy farmers, and the sharing of coupons in communities. One village chief described the need to share coupons, which may also result in diluting the programme’s effect, as being about ‘love’.

“*In some districts when you give a beneficiary the farm inputs*, *the beneficiary actually uses those inputs but in other districts they share*, *so if I get two bags of fertiliser then the village head will ask me to share*! *Maybe with three or more people*, *so you will not see the impact there*! *So that is the challenge we have*.*” (KII 07*, *District Council)*

“*There are some farmers who after receiving the coupons because they are too poor… they end up selling the coupons*, *because the coupon is not their immediate need*, *their immediate need is food*.*” (KII 08*, *District Council)*.

### Evaluation and reform

Nationally and globally the FISP has been widely considered a success, transforming Malawi from food insecure and hungry to self-reliant and a net food exporter [20]. As previously described, the FISP’s success has been largely described in terms of it being a smart subsidy [36], with the use of vouchers consistently emphasised as key to this. Far less attention has been paid to the political factors shaping FISP outcomes. Furthermore, whilst there is evidence of benefits from the FISP to agricultural production, the dietary and nutritional impacts are less clear, as previously described. Our previous quantitative work showed that in food-insecure populations of rural Malawi, maize was prioritised over more diverse foods independently of maize price [31, 32]–a finding likely to be related to the high levels of poverty and food insecurity in this population [127].

Community perspectives of the FISP impact on dietary diversity were mixed. Village chiefs were often positive, focusing on higher maize output, incomes, and legume promotion. However, others in the villages were generally negative about FISP impact on diets/nutrition, as agricultural output remained low, and food in the markets unaffordable. They described how it is hard from selling maize to be able to afford other foods–and even *ganyu* (casual farm labour, or agricultural piece work) on other peoples’ farms was in short supply. Some described how government export bans on maize had kept prices low, detrimentally affecting their livelihoods. Others mentioned how those who grew tobacco had better food security and potential for better nutrition.

“*FISP did not change [anything at local level] but maize price is very low this year*. *We are selling at K50 per kilogram (KG) so we are making a loss and cannot afford to buy a bag of fertilizer*.*” (FGD 5 of men*, *Phalombe District*, *post-harvest season)*

“*Those that cultivate tobacco are the ones that have more money*, *and they do buy more maize after selling their tobacco and they may also buy other foods like bread and the like*, *in addition to maize*.*” (FGD 3 of women*, *Lilongwe District*, *post-harvest season)*

Whilst many respondents perceived no gendered impacts, inequitable gender impact was described by several district-council and civil-society respondents. The FISP was described by one civil-society respondent as providing income to female farmers through cultivation and sale of legumes (especially in the southern region), some of which are considered female crops. However, the respondent was quick to note that it is often the male farmer who actually decides on the cropping, sells the product and collects the proceeds. These findings of the diverse ways in which women are disadvantaged have also been found in other work [128], and yet the presence of a woman in a household was also associated with greater household dietary diversity [32, 129, 130].

District-council respondents, despite expressing reservations, were often positive in general terms about FISP impact on diets/nutrition. National-government respondents from both health and agriculture expressed positive sentiments based on perceived causal pathways between the policy and better nutrition. Agriculture respondents had far greater awareness of the FISP than health respondents and were particularly positive. From the MoH, we were unable to speak to anyone particularly well informed about the FISP, and respondents often conceded that their views were not based on empirical policy evaluation. One MoH respondent did raise that Malawi’s focus on maize was addressing food security in terms of calorie adequacy but not supporting dietary diversification. Civil-society respondents mainly expressed reservations, describing how a focus on maize did not help to diversify diets due to maize being a low-monetary value crop, and expressing support for high-profit legumes that would raise household income and allow the purchase of other foods. However, such suggestions should be considered in the context of findings from Matita et al. (2021) that higher-value crops such as legumes are only prioritised when households have sufficient maize [32].

“*[The communities] have a better diet [due to the FISP]*… *The communities have what they need*. *They can either purchase or get food from gardens*. *So the overall general health of the population is improving*.*” (KII 10*, *District Council)*

“*The other big challenge in rural smallholder farming when it comes to maize production*, *is that maize is not a high value crop*. *So*, *they might have surplus in terms of the bags harvested*, *but… that income was not that much for them actually to diversify their diets*, *to have more money to buy other types of needs for the household*… *So that is the challenge with the staple food crop*. *So*, *when it comes to legumes for example they are high value food crops… within the same piece of land you can produce maybe same amount in terms of kilograms*, *but if you go to the market*, *you would get a lot more income*.*” (KII 20*, *non-governmental organisation)*

Other stakeholders, particularly from civil society but also village respondents, described minimal programme impact. Furthermore, district-council respondents often saw people as being ‘dependent’ on receiving the subsidy, with farmers waiting on coupons rather than planning themsleves on how to procure agricultural inputs.

“*If there were more maize harvests then it will be easily accessible and cheap but FISP does not satisfy this*.*” (FGD 1 of men*, *Lilongwe District*, *post-harvest season)*

“*Most people do not benefit*. *People are very poor*. *If you go to their fields you would not think that they were assisted in any way*.*” (KII 5*, *village head*, *Lilongwe District*, *lean season)*

Civil-society and village respondents often described how it was the ‘middle man’, intermediary between farmers and large traders, who made money from any increased agricultural production from the FISP. A civil-society respondent described how farmers would receive K40/kg for pigeon peas (a type of legume), but the government will buy pigeon peas from the middle man at K230/kg. People in the villages, from both districts, described how FISP had led to little change for them. Due to their ignorance of the optimal price for their crops, lack of negotiating power and financial pressue to reach a quick sale, they continue to sell their products to buyers at a lower price than can be obtained elsewhere.

“*What happens with us farmers especially from the rural [villages]*, *the buyer determines our prices*. *We don’t set the prices ourselves*… *The [buyer] can tell us that they would buy our maize say at K30*. *So*, *our selling of crops depends on what the buyer has instructed… and at the same time the crops are in abundance so they can leave to buy crops from somewhere [else]*.*” (FGD 11 of men*, *Lilongwe district*, *lean season)*

“*They [farmers] have to sell [immediately] so that they should have money*, *they can send their children to school and at least they have a relief on what to eat*.*” (KII 23*, *non-governmental organisation)*

Discussion of FISP improvements/alternatives from our interviews and FGDs was often held in the context of broader causal drivers of poor harvest, particularly climate change and weather variability. Each stakeholder group described possible improvements and alternatives, however people in the villages, local government and civil-society respondents were most likely to suggest alternatives to the scheme; respondents from government departments provided few views on this, largely endorsing the FISP.

FGD participants from the villages suggested alternatives, including more coupons; establishment of farmer clubs (for an agricultural input lending scheme); setting a universal fertiliser price; regulating maize and legumes price paid by vendors; and improvement on the timing of voucher distribution. Local-government respondents also suggested: more timely distribution of coupons; focus on productive farmers, or child-headed household targeting; a way to help beneficiaries graduate from the scheme; a separate programme for the poor (cash transfers); developing private-sector suppliers and banking services.

In regard to the suggestion of a cash transfer programme for the poor, at a national level Malawi elites have been described as tending to prefer policies that support the poor to work to support themselves, and in this way the FISP would be supported over a cash transfer programme [131]. In addition to the FISP’s role in helping to shore up the popularity and legitimacy of the government [36], this preference amongst elites for a programme in which the poor work to support themselves helps to explain the universal nature of the FISP. Several scholars have described how in other parts of Malawi’s agricultural sector, powerful interests have influenced government institutions and policies in such a way that only a minority of the population benefit. An example of this is tobacco policy, which, with its roots also in colonial agricultural policy, has increased dependence on a crop that only benefits a minority of Malawians [132]. Thus, the government implementation of the FISP (and AIP) is intended to appease the population whilst being palatable to the elites, and once implemented, a policy such as the FISP is politically difficult to remove.

Some respondents particularly from the health sector picked up on the differences in types of malnutrition being addressed, speaking about the need for the FISP, or any alternative policy/programme to support Malawians to diversify away from maize, to tackle dietary diversity. Comments related to this about crop diversification were also made in the context of need for greater resilience to environmental effects.

“*We think everything is maize but… food is not just maize; food is anything that gives us nutrients in the body*. *Of course*, *our staple is maize*, *but we have other foods like sweet potatoes*, *cassava*, *sorghum*.*” (KII 19*, *Ministry of Health)*

**“***FISP should target nutrition security not just the food security… maize is not equal to good nutrition but rather let us focus on nutrition security because when we focus at nutrition security*, *then we will be looking at diversity not just the maize*.*” (KII 19*, *Ministry of Health)*

However, such suggestions of the FISP’s focus on maize not addressing dietary diversity and nutrition should be considered in the context of work by [32]. This analysis suggests that addressing different types of malnutrition is one of complementarity rather than of competing agendas, with a need for dietary diversity and nutrition to be addressed alongside and perhaps secondarily to issues of hunger and food security.

Continuing on the theme of ‘love’ mentioned earlier in the context of the sharing of coupons, any changes to the programme as described by a village chief need to reflect the importance of sharing and community.

“*Firstly*, *its love*. *If we don’t have love*, *things will not work for us*. *For example*, *if I receive 10 coupons then I give 5 to my friends to share*, *then that is selfishness*. *If they are 6*, *we need to share equally*.*” (KII 6*, *village head*, *Lilongwe)*

The FISP was replaced in 2020 by the Affordable Inputs Programme (AIP) which has a focus on subsidies for maize seed and fertiliser but also includes sorghum and rice [28]. Legumes are not part of the AIP, suggesting a focus away from dietary diversity in favour of food security, although such interpretation should be taken cautiously given the complexity of impact pathways [32].

## Discussion

Our analysis was structured according to the Kaleidoscope Model (KM) for understanding policy change in developing countries. We found the KM very helpful for this analysis of the political economy of the FISP in regard to its impact on addressing malnutrition and the contextual factors affecting this. In particular, this analysis highlights the views of Resnick et al. (2018), the KM authors, that policy impact is dependent on policy and political processes and that understanding these processes is a critical part of understanding how best to achieve intended policy objectives. It also highlights the views of Resnick et al. (2018) and others that policy processes are much more complex, non-linear, and iterative than is often recognised. As such, the ‘360’ view of the conditions affecting policy change as provided by the KM is a most useful heuristic for addressing this complexity.

As is usual in the implementation of public policy, the adoption of Malawi’s FISP came about not just as a result of the external situation, such as that relating to a history of food shortages, but through a political process shaped by the interests, ideas and influence of the government and other stakeholder groups. Malnutrition is an issue of high political priority in the country but how it is conceptualised and the solutions for how to address it differ amongst stakeholder groups. Despite this range of positions held, AISP have a long history in Malawi with the FISP implemented over a 15-year period and recently replaced by the AIP, of a similar nature, albeit arguably more focused on food security. All major political parties support fertiliser subsidies–a policy approach difficult for a Malawian government to ignore, due to deep public concerns about food shortages and the production of maize being seen as key to addressing this. Accordingly, fertilier subsidy programmes are used by governments to shore up popularity and legitimacy [36].

Amongst the international community the FISP is considered to have played a significant role in improving maize production in Malawi in the context of population increases [26]. It has also maintained the commitment of policy champions within Malawi, with its recent replacement, the AIP, being of a similar nature. However, in-country stakeholders hold mixed views as to its design, implementation and impact. A number of stakeholders attribute the FISP’s success with agricultural production to favourable weather and other circumstances supporting the policy implementation, rather than to the programme itself. Others consider that it has had very little impact at all, and particularly with addressing nutritional concerns, a finding supported by previous studies [14, 28, 29, 133–135].

In terms of policy design, the FISP aimed to increase agricultural productivity and raise farmers’ incomes–and also to increase national food self-sufficiency [26, 72]. Legumes were also introduced, which may improve nutrition through dietary diversification based on own-farm production (or through higher incomes and more diverse food purchases) although the evidence is weak and suggests that the relationship between AISP targeting legumes and dietary diversification may only apply to food secure households [32]. Thus, the FISP’s food-related aims focussed primarily on addressing food shortages and hunger, and perhaps secondarily to dietary diversity and micronutrient malnutrition. This prioritisation given to addressing food security is understandable for a country with large proportions of the population facing food insecurity [9]. It is also perhaps unsurprising given the more visible nature of food shortages and hunger and high political support for addressing them, compared to a less visible and longer-term issue such as micronutrient malnutrition.

Another issue of policy design is the debate about programme efficiency. Reflecting an issue also discussed in prior literature [72, 78], several local government respondents held the view that the FISP is inefficient when those receiving coupons are poor farmers with limited agricultural production capacity. Rather, they advocated for the programme to target productive farmers, and for a different system needed to address poverty. These local government respondents tended to be optimistic about the programme, but blamed the lack of impact on the behaviour of the beneficiaries, while the rural people targeted by the programme tended to blame the nature of the programme. Others highlighted that rather than being a problem with policy design, instead the efficiency of the programme could be improved through better programme implementation.

The view that malnutrition is a problem of individual food choice fits with study findings from many contexts emphasising individual agency and attributing poor health to individual responsibility, often within a neoliberal paradigm, and neglecting structural influence shaping individual lives [49, 136–138]. In contrast, as other literature has found [66], respondents in rural villages pointed to a different pathway of causation, the unaffordability of diverse, healthy foods rather than due to their choice. This resonates with repeated international calls to make healthy diets affordable [139].

There was also considerable discussion relating to the politics of targeting. While there was some agreement that identification of beneficiaries at central level was an improvement over the previous selection of beneficiaries by chiefs, perceptions of patronage and corruption remains. There was also mention from the village and civil-society respondents of capture of the profit margin in the value chain, with the ‘middleman’ perceived to be benefitting the most.

Related to the issue of policy efficiency is the issue of crowding-out of the private sector, which can occur where farmers receive the AISP rather than purchasing the inputs privately, at market price. With current programme design, targeting poor farmers who cannot afford fertiliser from the open market, this is less of an issue. However, it would be a significant political/economic concern if the programme, as suggested by many district-level respondents, was redesigned to target productive farmers.

The respondents raised several issues relating to policy implementation, and the dynamics of top-down and bottom-up policymaking, that may also help explain the lack of nutritional impact. Delays in programme implementation were often described, with late issuing of coupons a particular problem with direct impacts on the agricultural production potential of beneficiaries. Other problems included its poor targeting of beneficiaries, a perceived lack of coupons, problems with policy coordination, coupon sharing and selling on, and the conflict it has brought to communities. Our analysis also points to the way that on-the-ground decision-making amongst the people targeted by a policy critically influences its impact, given the redistribution of coupons at community level. The reportedly common practice of sharing and selling-on of coupons reflects the poverty experienced by those receiving the coupons, who sell the coupons to satisfy immediate financial needs, but also reflects the priority given by the rural communities to maintaining social cohesion in a context of severe poverty and food and nutrition insecurity, with little in the way in form of social safety nets. Whilst targeting criteria have been developed to support identification of beneficiaries, the sharing of coupons suggest that such criteria are ignored for the maintenance of social cohesion.

Some of this on-the-ground shaping of policy in the form of the sharing/selling-on of coupons, as also described elsewhere [140, 141], is driven by policy design, about there being a limited number of coupons. However, it also reflects tensions between top-down and bottom-up policy implementation dynamics–and particularly exemplifies bottom-up understandings of policy implementation, which emphasise that policy implementers alter the policy implementation process. This dynamic would be lost in a model that was simply based on top-down understanding of policymaking, and thus the KM also helps us to understand this dynamic, through its identification of ‘implementation veto players’ as shaping policy implementation. However, in this case, rather than it being the policy implementers who change the policy in its implementation, it is the recipients of the policy themselves. Communities are using the programme in the way they think fits best with their desires which include solidarity, provision of economic support, and social cohesion–important for the community but not necessary for the programme designers (government). This is an important extension to current models of policy implementation including the KM–and an important consideration for policymakers in sub-saharan Africa and other regions considering AISP and other policy impact at the community level.

The FISP’s sustainability over time is maintained by the political mileage it provides governments, and its universal nature makes it is less of a political risk. The Malawian public have for a long time been accustomed to government intervention to increase maize productivity with proposed income and dietary benefits, with maize also featuring as a dominant product in Malawian diets and dietary discourse. Thus, the FISP’s nature and its focus on maize reflects institutional values and norms of behaviour to which the public have grown accustomed. The FISP is being implemented in a low-income country with high levels of poverty, food insecurity and malnutrition. Thus, it is considered an appropriate use of limited funds by stakeholder groups including the public, given its potential to address these pressing issues. Whilst it is implemented by the Malawian government, it has the support of other actors within the country including donor agencies. Thus, the FISP is considered an appropriate response to addressing Malawi’s problems of poverty, food insecurity and perhaps indirectly also malnutrition, whilst also shoring up public support for the government and thus decision makers’ jobs.

The study respondents suggested numerous improvements to FISP implementation. Whilst there is scope for the FISP, and its replacement the AIP, to be more effective, including through implementation of complementary actitivities, any expectations here need to be considered in the context of the findings above that anything similar to the FISP’s current form is unlikely to markedly improve dietary diversity. This is also supported by evidence from studies on crop diversification that the inclusion of legumes is not necessarily resulting in nutrition-sensitive consumption, especially given the need for poor and food-insecure households with little land (approximately 0.3 hectares) to prioritise production of maize ahead of other crops [32, 107, 142, 143]. Several authors have discussed the need for complementary nutrition extension programmes to support behaviour change in the eating habits of Malawians [29, 144]. However, as reflected in comments from our village respondents, unless paired with policy/programmes that more fundamentally change the context in which people make their food choices, it is unlikely to have much impact.

Our analysis has several limitations. These include that the interviews were particularly geared towards village respondents and chiefs, with fewer people interviewed at Ministry levels who might know the policy better, and as with other study methodologies, there is likely a tendency for participant bias in responses provided–for example with some stakeholders who perceive benefit from the programme as it runs currently to provide supportive responses, and those in villages with need for government support to downplay any benefit that they may receive. However, even with these limitations, our findings are supported by the literature on these topics more broadly, and also by our work using other, quantitative, methods [28, 32]. The competing elite-stakeholder narratives also reflect findings of others in nutrition and agricultural policy [10, 145], however our study extends analysis to the beneficiaries of the programme, who we have identified as having a different narrative regarding the programme effects to policy implementers and through their actions change the policy from what was initially intended.

## Conclusion

The Kaleidoscope Model (KM) is useful for helping to understand the ‘gap’ between policy expectations and outcomes with agricultural subsidy programmes in Malawi. This case study also highlights that the KM, and other models of policy implementation, could usefully be extended to include that the people targeted by the programme themselves shape the policy process–an extension of the concept of ‘implementation veto players’.

Our findings add to existing evidence of AISP impact on dietary diversification and nutrition, and provide new insight into the political economy of AISP impact. Barriers to impact relate, in this case study of Malawi’s FISP, to issues of policy design, policy implementation, and characteristics of the target population in a context of subsistence farming marked by extreme poverty, seasonality and food insecurity.

With policy design, the Malawi case study raises issues of targeting and efficiency, as well as the aims of AISPs and the contradictions/challenges with addressing both food security as well as dietary diversity. With policy implementation, the data highlight a range of issues contributing to and also detracting from policy implementation, including the way that on-the-ground decision-making amongst the people targeted by a policy influences policy impact, in this case resulting in the sharing and selling-on of coupons, which is thought to dilute programme effect.

Whilst reform of the FISP, or its replacement the AIP, would be partially beneficial, addressing important components of policy design and implementation, this study suggests that policy would be more nutrition sensitive if focused more directly on increasing smallholder farmer income, and mitigating impacts of seasonality and extreme climatic events. This could be achieved through social-assistance programmes and investment in smallholder irrigation [143, 146–148]. A considerable body of existing evidence documents a positive relationship between social assistance programmes and improved nutritional outcomes [149–152]. However (and especially given the suggestion documented above that even when maize prices are considerably low rural people still maintain a preference for maize over more diversified foods [31]), it is worth noting the findings of Hoddinott et al. (2014) that contrary to the findings in middle-income settings, cash transfer impact may not result in dietary diversification in settings where income is much lower and food insecurity is common due to fluctuating prices [153]. However given that subsidy programmes such as the FISP are likely to continue to be a major feature of development policy in many countries in Africa for a variety of political and other reasons, committed effort should be made to improve their effectiveness and efficiency [154], including in regard to improving nutrition through dietary diversification. However, this would require that nutrition is deliberately set as an objective at the design stage of the policy process. It is important for policymakers to be explicit about the desired policy outcome–perhaps in regard to nutrition as well as food security–and to take into account beneficiaries views in a more bottom-up policymaking approach. These are issues that apply widely, to policy-making in Malawi and also elsewhere.

More broadly, this analysis highlights the importance of food systems that deliver both adequate quantity as well as quality and diversity of foods, the interlinkages between these different food system outcomes, and the challenge for food policy actors to find ways to improve the compatibility of healthy nutrition with food security [9, 32, 155].
